# Biophysical Factors Affecting the Distribution of Demersal Fish around the Head of a Submarine Canyon Off the Bonney Coast, South Australia

**DOI:** 10.1371/journal.pone.0030138

**Published:** 2012-01-11

**Authors:** David R. Currie, Sam McClatchie, John F. Middleton, Sasi Nayar

**Affiliations:** 1 South Australian Research and Development Institute (Aquatic Sciences), Adelaide, South Australia; 2 Southwest Fisheries Science Center, National Oceanic and Atmospheric Administration (NOAA) Fisheries, La Jolla, California, United States of America; University of Western Ontario, Canada

## Abstract

We sampled the demersal fish community of the Bonney Canyon, South Australia at depths (100–1,500 m) and locations that are poorly known. Seventy-eight species of demersal fish were obtained from 12 depth-stratified trawls along, and to either side, of the central canyon axis. Distributional patterns in species richness and biomass were highly correlated. Three fish assemblage groupings, characterised by small suites of species with narrow depth distributions, were identified on the shelf, upper slope and mid slope. The assemblage groupings were largely explained by depth (*ρw* = 0.78). Compared to the depth gradient, canyon-related effects are weak or occur at spatial or temporal scales not sampled in this study. A conceptual physical model displayed features consistent with the depth zonational patterns in fish, and also indicated that canyon upwelling can occur. The depth zonation of the fish assemblage was associated with the depth distribution of water masses in the area. Notably, the mid-slope community (1,000 m) coincided with a layer of Antarctic Intermediate Water, the upper slope community (500 m) resided within the core of the Flinders Current, and the shelf community was located in a well-mixed layer of surface water (<450 m depth).

## Introduction

Abrupt submarine topographies such as canyons, seamounts and shelf-breaks are becoming increasingly recognised as key hotspots of productivity in the oceans [1], [2], often vitally important to sustaining fish production. Studies in the Mediterranean, Georges Bank, and off the Oregon and Canadian west coasts have shown that canyons generate complex flows, the net result of which can be higher regional productivity [3], [4], [5], [6]. While smaller zooplankton and phytoplankton may be advected offshore by temporally variable flows generated around canyons, swimming and vertically migrating micronekton such as krill and mesopelagic fish can maintain position within canyons by behavioural interaction with the flow field [4], [5], [7]. These aggregations of micronekton are preyed upon by commercial species, such as *Sebastes* on the North American west coast [8], and provide a rich food source for cetaceans [9]. As a result, Astoria Canyon off Oregon is an important fishery area with extensive groundfish dependent upon the rich prey field of the canyon [8]. Similar processes may also enhance fisheries around canyons off South Australia, but have yet to be investigated.

The processes that affect upwelling within canyons are known to include tidal mixing and rectification as well as large-scale along-slope currents [10], [11]. In the context of the latter, the shoreward pressure gradient that is normally balanced by geostrophy, may be ruptured within narrow canyons resulting in up-slope transport of fluids [12]. Off South Australia two sources of deepwater current may contribute to upwelling. The first, known as the Flinders Current, flows from east to west at depths of 400–800 m with speeds of 5–10 cm/s, and is driven by the on-shore Sverdrup transport from the Southern Ocean [13]. In the western Great Australian Bight, the upwelling can be in the order of 200 m over an offshore distance of 50 km [14]. This current is generally strongest during summer, but can be non-existent or reversed by winds and thermohaline circulation. Secondly, westward slope currents associated with warm core eddies may also lead to upwelling within the canyons of the region [15]. In addition, wind-forced upwelling closer to shore can raise water and nutrients from depths of 150 m or so to near the coasts of Kangaroo Is and Robe [16], [17], [18], [19], [20], [21]. As for the region's fisheries, the roles of canyons as paths of nutrient and sediment upwelling between the deep-slope and coast off South Australia remain to be determined.

The continental margin off South Australia is cut by numerous massive submarine canyons, many of which drop steeply to depths of 5 km, with walls up to 2 km high [22]. The Bonney Coast canyons are of particular importance because the area is being explored for hydrocarbons [23]. Tar balls stranding along the Bonney coast may be transported up the canyons from natural leaks at the base of the slope. If proven, this may indicate oil-bearing sediments buried in up to 4000 m deep water, in this un-drilled region. During summer, westward currents over the slope generated by the Flinders Current and mesoscale eddies could produce currents within the canyons sufficient to move sediments and nutrients up slope [24]. Before a need arises to manage hydrocarbon extraction, it is imperative to understand the importance of the canyons to regional productivity.

**Figure 1:**
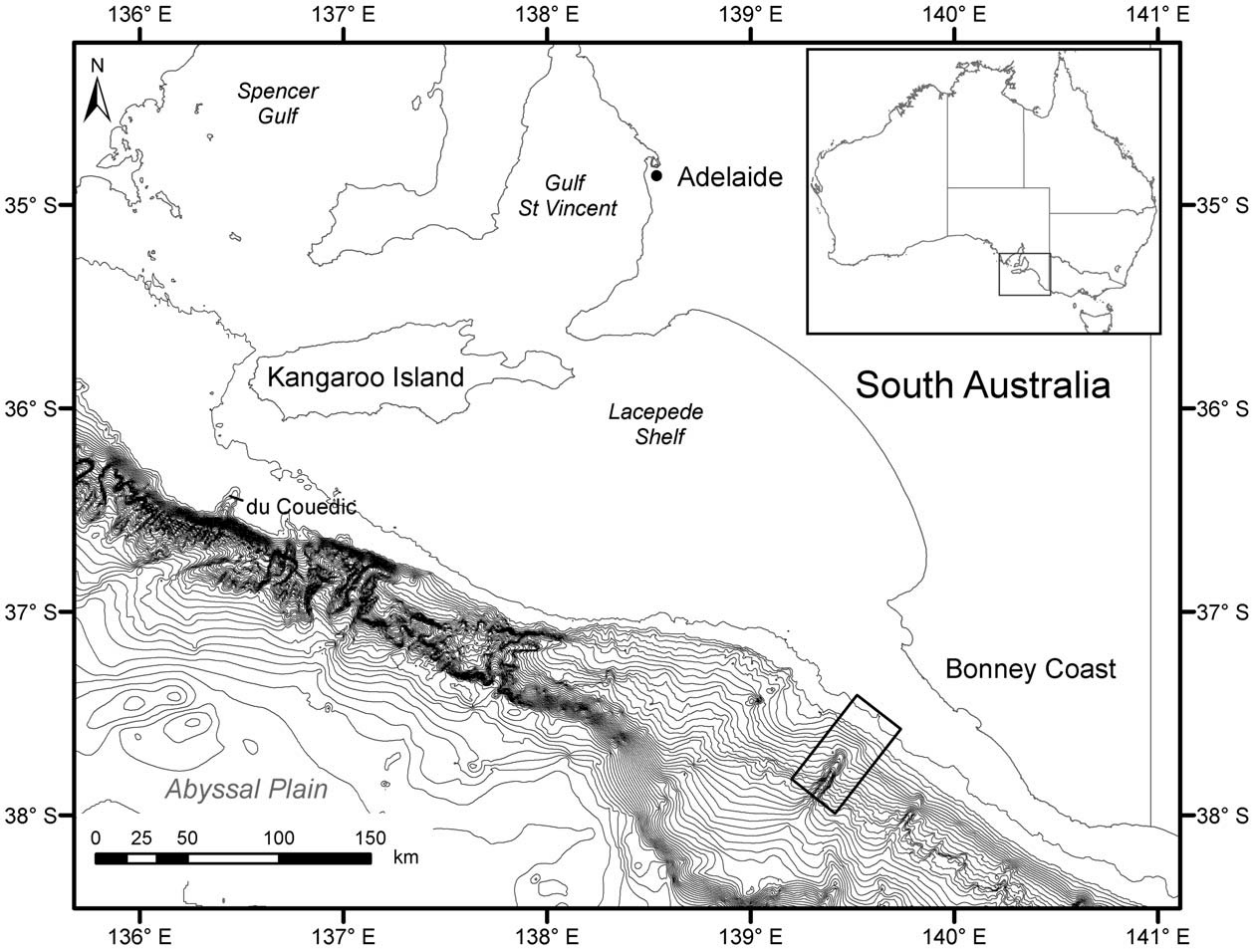
Map showing the location of the study area (unfilled rectangle) on the south-east Australian continental slope. Contour lines presented follow 100 m depth intervals.

As part of a multidisciplinary study conducted during February 2008, physical, chemical and biological measurements were collected from two canyon systems (Bonney and du Couedic) to evaluate their geological and hydrodynamic settings and their roles in enhancing marine productivity. In this paper we concentrate on oceanographic and trawl data collected at Bonney Canyon (Figure 1) during the first leg (4–16 February 2008) of voyage SS02/2008 on the Australian National Facility RV *Southern Surveyor*. In particular, we examine the composition and distribution of demersal fish in relation to canyon orientation and topography, to determine whether discrete hotspots of biomass and diversity exist. Additionally, we evaluate the relative contributions of ambient environmental conditions to observed spatial patterns, in an effort to determine the dominant factors structuring demersal fish communities. This assessment is guided by a conceptual model that evaluates the occurrence of canyon upwelling, the magnitude and direction of near-bottom currents, and the likely mixing paths for nutrients and marine biota.

## Materials and Methods

An ethics statement is neither issued nor required to undertake demersal fish trawling either commercially or for research in Australian waters. In South Australia (SA), the legislation covering animal welfare is the Prevention of Cruelty to Animals Act 1995 and Prevention of Cruelty to Animals Regulations 2000. The Prevention of Cruelty to Animals Act (POCTA) specifically excludes fish.

### Survey design

Initially, a broad-scale mapping survey of the target area was conducted using multi-beam sonar (Kongsberg-Simrad EM300) and an onboard Acoustic Doppler Current Profiler (ADCP) to obtain high-resolution bathymetry and a synoptic picture of the canyon and coastal upwelling. These data, together with real-time sea surface temperature (SST) imagery obtained from the National Oceanic and Atmospheric Administration (NOAA) satellites, were used to resolve the upwelling front, and guide our detailed sampling of the area (Table S1).

**Figure 2:**
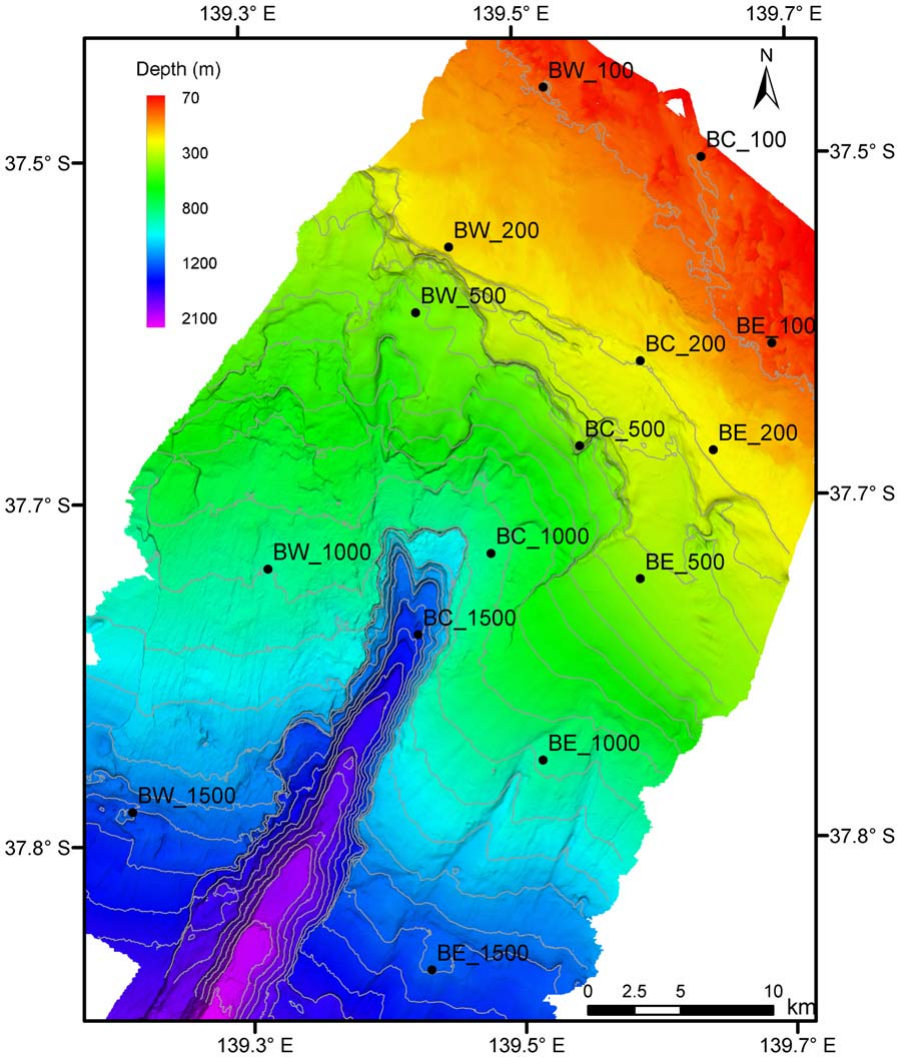
Bathymetric map of Bonney Canyon showing the locations of 15 depth-stratified sampling sites. Labels denote location relative to the central canyon axis (BW = Bonney West, BC = Bonney Centre, BE = Bonney East) and sampling depth in metres (100, 200, 500, 1000, 1500).

In order to capture both the canyon feature and upwelling event, three parallel transects (approximately 10 km apart) were established along, and to either side of the central canyon axis. Five depth-stratified sampling stations (100 m, 200 m, 500 m, 1000 m and 1500 m) were then established along each transect and used to measure water column and seabed properties, and to quantify the composition and distribution of demersal fish (Figure 2).

### Oceanographic measurements

Profiles of water temperature, salinity and pressure were recorded at each sampling site using a Seabird SBE911 CTD fitted with modular sensors for dissolved oxygen (Aanderaa Optode 3975) and fluorescence (Chelsea AQUAtracka). All of these instruments were attached to the vessels 24-bottle rosette frame, and lowered to within 20 m of the seabed during each cast. Seabird-supplied calibration factors were used to compute pressure and temperature, and their accuracy validated over the course of the voyage by examining, and correcting, deviations in the sensor records before and after each cast [25]. A series of 18 Niskin bottles mounted on the rosette frame were used to collect water samples at up to 6 depths on each cast. At the very least, these collections included samples from the surface, the seabed, and the depth of maximum fluorescence. These samples were primarily collected to estimate spatial variability in phytoplankton productivity, but were also used to calibrate the salinity, oxygen and fluorescence sensors. As this was primarily a demersal fish study, CTD data extracted from deepest part of each vertical cast were utilised in all subsequent analyses. The CTD profile data from the overlaying water column were incorporated separately in a conceptual hydrodynamic model.

### Sediment sampling

The composition and structure of the seabed at each of the 15 depth-stratified sampling sites was determined from replicate 0.1 m^2^ Smith-McIntyre grab samples. Two sediment sub-samples (70 ml and 10 ml) were collected from each grab by scraping an open vial across the top of each sample. These were snap-frozen and stored at −20°C before being analysed. The larger of the two sediment sub-samples was wet-sieved through an agitated stack of Endicott sieves to determine the grain-size structure and sorting coefficients of the sediments. The smaller sample was dried and ground to a talcum-powder consistency before being processed in an elemental analyser (Europa Scientific ANCA-SL) coupled to a mass spectrometer (Geo 20-20) to determine organic carbon and total nitrogen content.

### Trawl sampling

A single trawl shot of 40 minutes duration was conducted at each site using a Kavanagh otter trawl. This net had a headline length of 21.6 m and a headline height of 3.5 m, and was fitted with a 50 mm mesh cod-end. The net was towed at a speed of 3 knots using 50 m bridles and polyvalent doors. Scanmar transducers mounted on trawl doors and net were used to monitor the height, spread and orientation of the trawl gear, and ensure consistent ground contact between shots. Repeat trawl shots were necessary at two sites due to gear malfunctions including a seabed hook-up (BC_100) and overspreading of the net (BE_500). Due to the extremely rugged nature of the seafloor at 1500 m, planned trawl shots at this depth were not undertaken.

The entire catch from each trawl shot was processed onboard and each component taxa identified to species (using the diagnostics of Gomon *et al.* [26]) before being counted and weighed. Voucher specimens of all species collected were preserved in 70% ethanol and subsequently lodged with the South Australian Museum, Adelaide. Small samples of muscle tissue were also dissected from each species and archived for genetic analyses, while the remainder of the catch was discarded overboard.

### Data analysis

Geographical information software (ArcGIS [27]) was used to characterise and display spatial trends in environmental data. Physical, chemical and biological attributes for each sampling station were interpolated using a kriging algorithm [28] and a series of maps was constructed. These maps were used to visualise discontinuities within the region and highlight patterns of similarity between variables. Relationships between each environmental variable were subsequently tested using Pearson correlations after confirming bivariate normality [29].

Prior to all analyses, measures of fish abundance and biomass were standardised as either number (n) or weight (g) per area trawled (hectares, ha). The area swept by each shot was estimated from the total distance trawled (obtained from GPS coordinates), and the average door spread.

Two-way fixed factor analysis of variance (ANOVA) was used to test for differences in the number of demersal fish species (richness) represented both along and either side of the central canyon axis and among different depth strata. Similar tests were also applied to examine depth and canyon-related differences in the standing-stock (biomass) of the fish fauna. Before conducting these analyses, homogeneity of variance was examined using Levene's test and heterogeneity removed where necessary by log10(n+1) and √(n+1) transformations.

Depth and canyon-related differences in fish community structure were also examined using Bray-Curtis (B-C) dissimilarity measures [30]. A single square-root transformation was applied to the data before calculating the B-C dissimilarity measures. This transformation was necessary to prevent a small number of large species unduly influencing the B-C measures [31].

The computer package PRIMER was used to generate B-C dissimilarities and to undertake all multivariate analyses [32]. Spatial patterns in dissimilarity were initially mapped using a combination of hierarchical agglomerative clustering and non-metric multi-dimensional scaling (MDS), and depth and canyon-related differences tested using a two-way permutational analysis of variance (PERMANOVA) [33]. A similarity profile (SIMPROF) test was used to discriminate significant clusters, and the similarity percentages (SIMPER) routine of Clarke and Gorley [32] was subsequently used to identify those species contributing most to observed differences. The extent to which measured environmental variables explained our community patterns was tested using the biological environmental (BIOENV) routine of Clarke and Ainsworth [34].

### Water circulation model

The CTD data from the 15 sampling sites were examined in greater detail to provide a conceptual model that investigates the occurrence of canyon upwelling, the strength and direction of near-bottom ocean currents and the mixing paths for nutrients and marine biota.

A condition for the existence of canyon upwelling (or downwelling) is given by the ratio of the canyon width (*W*∼3 km) to the internal deformation radius a*_i_* [12]. The latter scale is that which the alongshore velocity (*v*) can “deform” into the canyon. Where *W* is small compared to a*_i_*, the velocity cannot divert into the canyon where *v* is then zero. In this case, the onshore pressure force that would normally be balanced by the Coriolis force acts instead to accelerate fluid up the canyon and towards the coast. The deformation radius is defined by a*_i_* = NH/f where the buoyancy frequency N is given by
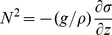
(1)g = 9.8 m/s^2^ is the acceleration due to gravity, f = −8.9×10^−5^/s, is the Coriolis parameter, σ the potential density of sea water and ρ the average density of sea water (1026 kg/m^3^). Estimates of N^2^ were obtained for all 15 sampling sites and for depths 200–1200 m, and indicate mean values of about N = 0.5×10^−5^/s. With H a scale for the water depth of 500–1000 m, a*_i_* is of order 13–26 km and much larger than the canyon width (*W*∼3 km). In this case canyon upwelling can occur (e.g. [12]). Now, if the alongshore velocity at the immediate top of the canyon is directed to the N.W., then upwelling will occur. During the survey, surface currents above the canyon were about 0–10 cm/s N.W., and favorable for upwelling.

To estimate the currents nearer the sea floor and at the rim of the canyon, we have calculated the thermal wind shear that gives the change in alongshore velocity with depth, and which arises from the slope in the measured density field. In particular, if the x-axis is directed offshore (along the central site) and the y-axis is positive to the S.W., then the thermal wind velocity at a depth *z* = −*h* (positive upwards) is
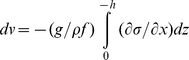
(2)


## Results

### Seafloor environment

Multi-beam soundings of the Bonney Canyon (Figure 2) reveal a diffuse entrance at the shelf-break (∼200 m depth) and a well-defined headwall on the upper slope (∼800 m depth). Below the headwall, the canyon is narrow (∼3 km wide) and deeply incised (>1 km deep) with steep sidewalls (gradient>1∶1). The floor of the canyon is terraced and bears numerous scallop-shaped scars that are indicative or erosion and slumping.

Samples of sediment taken around the canyon were variable in structure and ranged from silt, to coarse-sand and gravel. These sediments were found to be composed almost entirely of biogenic material, including fragments of sponges, bryozoans, molluscs, coralline algae and foraminifera. Spatial patterns in grain-size were broadly consistent with patterns in bathymetry. Sediments were typically coarsest in the shallow shelf waters, but became progressively finer with increasing depth and distance offshore (Table S2). Sediment sorting, by comparison, was less clearly related to depth, and was greatest around the shelf-break (Table S2). This observation is consistent with enhanced near-bed flow and scouring of sediments around the shelf-break.

The organic carbon content of the sediments around Bonney Canyon broadly reflected trends in sediment size structure (Table S2). Notably, organic carbon content was found to be lower in the coarser sediments of the shelf than in the muddier sediments of the slope. In particular, organic carbon was concentrated in those sediments occurring at a depth of approximately 1000 m on the central canyon axis. A similar distribution pattern was also observed for concentrations of sedimentary nitrogen (Table S2).

Marked depth-related differences in near-bottom water temperatures were observed around the Bonney Canyon (Table S2). A band of cool water (9.5–12.1°C) characterised the continental shelf inshore from the head of the canyon. Beyond the shelf-break, near-bed water temperatures gradually declined with increasing depth, and reached a uniform minimum (2.7°C) both on and either side of the central canyon axis at 1500 m depth.

Patterns in salinity were similar to those of temperature, and were generally higher on the shallower near-bed waters of the shelf, than on the deeper waters of the slope (Table S2). However, salinity did not vary directly with depth and was lowest (34.4) at the 1000 m depth stratum. Dissolved oxygen concentrations, by comparison, were much more tightly matched to temperature (Table S2), and were highest (>250 µM/l) on the shelf and lowest (171 µM/l) at 1500 m depth. Distribution patterns in near-bottom chlorophyll concentrations were also elevated on the shelf, but were notably highest (>13 µg/l) at the shallowest survey sites (100 m depth) located on and to the east of the central canyon axis (Table S2).

### Water circulation model

**Figure 3:**
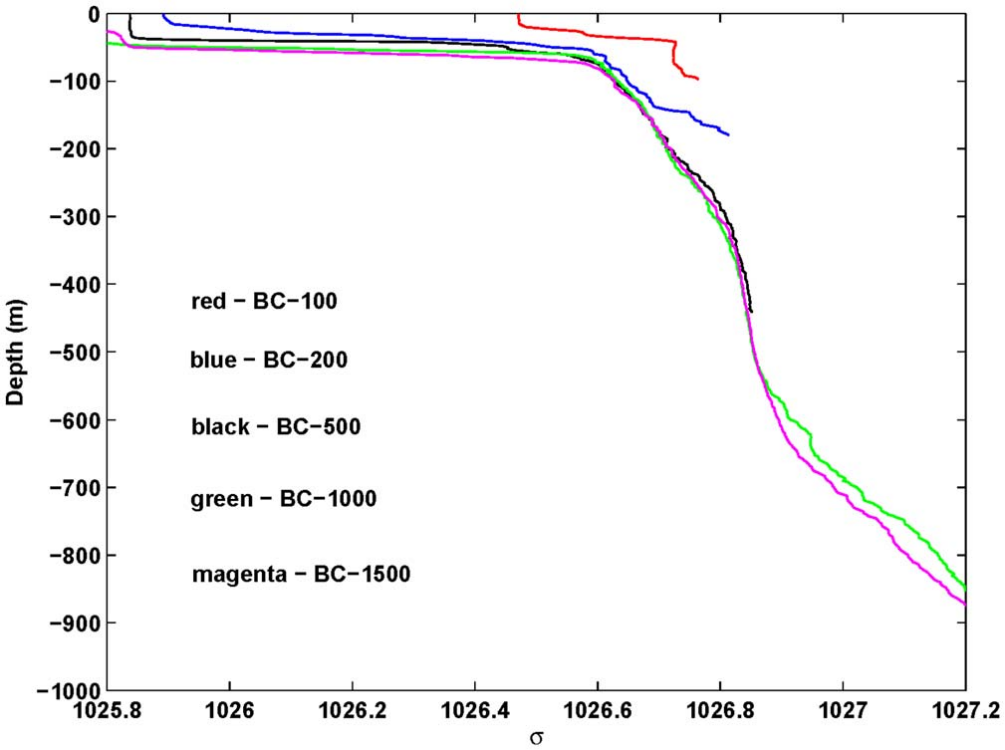
Depth (m) versus potential density (kg/m3) for the 5 central axis sites BC_100 to BC_1500.

For the central canyon axis, the potential density is plotted as a function of depth in Figure 3. The density profiles at sites BC_100 and BC_200 show the existence of a strong upwelled layer near the bottom, and a surface mixed layer (SML) about 50–75 m. The upwelled layer and SML are likely the result of strong S.E. winds which persisted from mid January to mid February. At depths of 500 m or so, the densities at the three offshore sites (BC_500, BC_1000, BC_1500) are very similar (∼1026.8 kg/m^3^) indicating that the isopycnals (and isotherms) are flat and that little upwelling has occurred. At greater depths of 600–900 m, the water at the BC_1000 site is denser than at BC_1500 (above the canyon gallery) indicating that upwelling has occurred.

**Figure 4:**
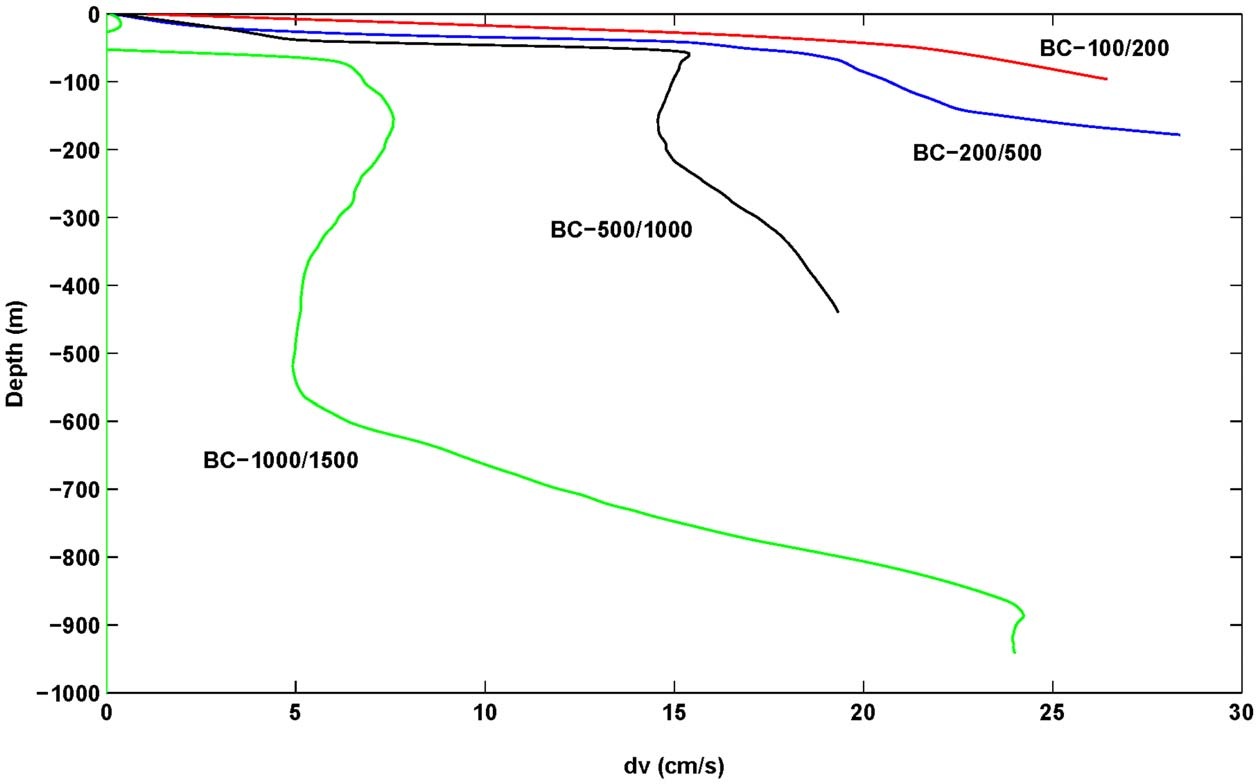
The thermal wind (current) in the alongshore direction, dv (cm/s) calculated from the CTD data and for site pairs BC_100–200, BC_200–500, BC_500–1000, BC_1000–1500. The positive values oppose the (negative) surface values that are to the N.W. leading to smaller net currents at depth.

Results for the thermal wind shear are shown in Figure 4 for each of the four adjacent CTD site pairs. For the shelf/upper slope sites (BC_100–200, BC_200–500), the thermal wind shear increases to around 27 cm/s at the bottom and is directed to the S.W. and opposite to the surface currents that are of order 30–40 cm/s. That is, the effect of upwelling is to reduce the total near-bottom currents to be 13–26 cm/s. This reduction in alongshore current speed is known as thermal wind shut-down [35], [36].

Over the canyon (site pair BC_1000–1500), the deep upwelling from 600–900 m (Figure 3), leads to a thermal wind shear at the canyon rim (∼1000 m depth) that is near 25 cm/s. Since the surface currents here are smaller (∼0–10 cm/s) the net alongshore current at the canyon rim will be around 15–25 cm/s and directed to the S.W. (i.e. the currents will act to downwell water within the canyon). This result pertains to the time at which the CTD casts were made. At earlier times, upwelling may have occurred in the canyon due to the processes described. However, we have no CTD pairs within the lower canyon, so we cannot determine if upwelling did occur.

**Figure 5:**
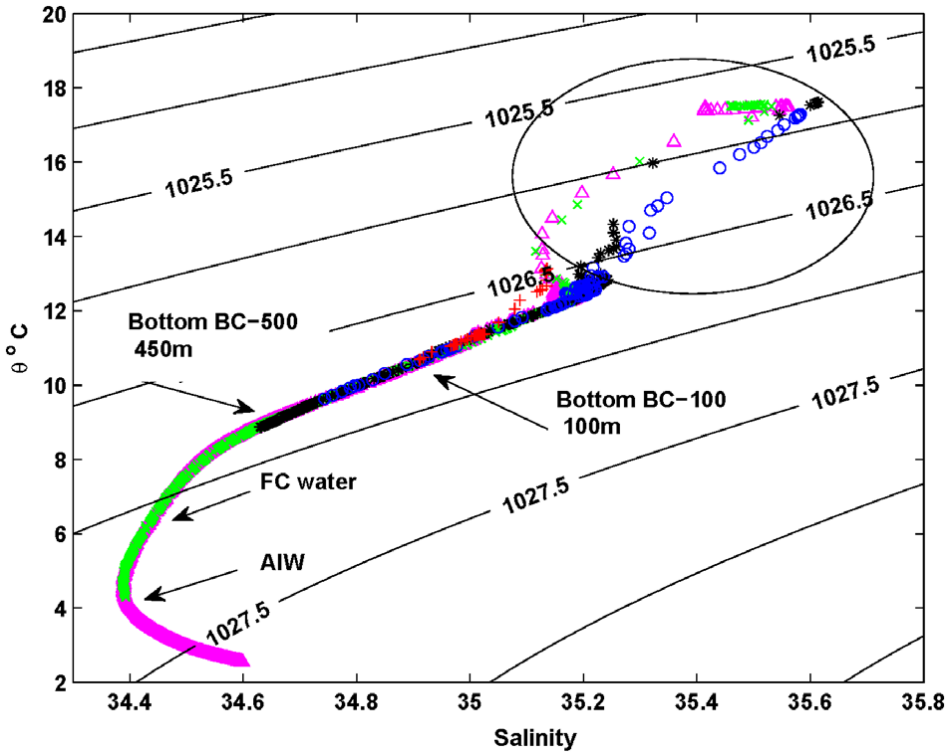
Potential temperature versus salinity for sites BC_100 (red cross), BC_200 (blue circle), BC_500 (black asterisk), BC_1000 (green cross), BC_1500 (magenta triangle). The potential density is contoured (units kg/m3). Flinders Current is denoted (FC) and Antarctic Intermediate Water (AIW).

To evaluate possible pathways for water mixing, a plot of (potential) temperature versus salinity was constructed using all central axis CTD data (Figure 5). Beginning in the canyon, the line of magenta triangles indicate mixing between abyssal waters (1500 m, 2.5°C) and the Antarctic Intermediate Water mass (1000 m, 4°C). Evidence for the upwelled bottom boundary layer of the Flinders Current is here shown in Figure 3 at depths of 600–900 m. We note also that at depths of 450–500 m, the water contains an oxygen maximum that is an indicator of Subantarctic Mode Water that is generally well mixed in the vertical.

Within the SML (i.e. 50–75 m from the surface and largely enclosed by the ellipse shown in Figure 5) the effects of heating and evaporation are evidenced by the scatter in the CTD data. However, between the base of the SML (∼100 m) and the top of the Flinders Current water (450 m), there exists a remarkably straight “mixing” line that encloses the data from all sites. The potential density along this line is not quite constant and varies from about 1026.8 to 1026.65 kg/m^3^. Notably, this mixing line includes the near-bottom data from sites BC_100 and BC_200 where strong upwelling is evident (Figure 4), as well as the upper depth water data from the offshore sites BC_1000 and BC_1500.

### Faunal composition

In total, 78 fish species from 46 families were collected from the 12 depth-stratified trawls undertaken in this study (Table 1). Of these families, Macrouridae (whiptails) were by far the best represented (11 species). The Moridae (cod) were the next most speciose family (4 species), followed by Neosebastidae (gurnard perch), Platycephalidae (flathead), Synaphobranchidae (basketwork eel) and Triglidae (searobin) (3 species each). A further 24% of families (11/46) were represented by two species, however, the majority of families (63%, 29/46) were represented by just one species.

**Table 1:** Mean abundance (n per ha ± s.e.), biomass (g per ha ± s.e.) and frequency of occurrence (n sites at which present, with % in brackets) of demersal fish from 12 depth stratified sampling sites around Bonney Canyon.

Family	Common Name	Scientific Name	Occurrence	Abundance	Biomass
Acropomatidae	Threespine Cardinal Fish	*Apogonops anomalus*	4 (33.3)	0.275±0.240	12.3±9.6
Alepocephalidae	Southern Slickhead	*Alepocephalus* sp.	1 (8.3)	0.004±0.004	1.0±1.0
	Sparkling Slickhead	*Rouleina squamilatera*	1 (8.3)	0.050±0.050	13.2±13.2
Apogonidae	White Cardinalfish	*Epigonus denticulatus*	1 (8.3)	0.008±0.008	0.4±0.4
Callionymidae	Painted Stinkfish	*Eocallionymus papilio*	1 (8.3)	0.008±0.008	0.6±0.6
Callorhinchidae	Elephant Shark	*Callorynchus milii*	2 (16.7)	0.013±0.009	28.8±22.9
Carangidae	Jack Mackerel	*Trachurus declivis*	3 (25)	0.179±0.145	24.7±14.4
Centrolophidae	Spotted Trevella	*Seriolella punctata*	1 (8.3)	0.004±0.004	7.6±7.6
Centrophoridae	Brier Shark	*Deania calcea*	1 (8.3)	0.025±0.025	71.0±71.0
Cheilodactylidae	Jackass Morwong	*Nemadactylus macropterus*	3 (25)	0.246±0.148	196.6±116.8
Congridae	Conger Eel	*Bassanago* sp. 1	4 (33.3)	0.046±0.026	29.5±17.9
Cyttidae	Silver Dory	*Cyttus australis*	1 (8.3)	0.004±0.004	1.4±1.4
	King Dory	*Cyttus traversi*	3 (25)	0.100±0.062	41.5±27.5
Dalatiidae	Plunkets Shark	*Centroscymnus plunketi*	1 (8.3)	0.004±0.004	4.4±4.4
	Black Shark	*Dalatias licha*	1 (8.3)	0.004±0.004	3.2±3.2
Diodontidae	Australian Burrfish	*Allomycterus pilatus*	4 (33.3)	0.029±0.013	28.2±13.8
Emmelichthyidae	Redbait	*Emmelichthys nitidus*	3 (25)	0.521±0.345	111.1±88.1
Euclichthyidae	Eucla Cod	*Euclichthys polynemus*	3 (25)	0.163±0.102	5.7±3.7
Gempylidae	Gemfish	*Rexea solandri*	1 (8.3)	0.004±0.004	4.5±4.5
	Barracouta	*Thyrsites atun*	1 (8.3)	0.029±0.029	6.7±6.7
Heterodontidae	Port Jackson Shark	*Heterodontus portusjacksoni*	1 (8.3)	0.004±0.004	2.6±2.6
Hexanchidae	Broadnose Seven Gill Shark	*Notorynchus cepedianus*	1 (8.3)	0.004±0.004	0.8±0.8
Hoplichthyidae	Deepsea Flathead	*Hoplichthys haswelli*	1 (8.3)	0.017±0.017	4.4±4.4
Idiacanthidae	Black Dragonfish	*Idiacanthus atlanticus*	2 (16.7)	0.017±0.013	0.3±0.2
Macroramphosidae	Banded Bellowfish	*Centriscops humerosus*	2 (16.7)	0.204±0.138	11.5±7.8
Macrouridae	Southern Whiptail	*Caelorinchus australis*	1 (8.3)	0.021±0.021	7.8±7.8
	Notable Whiptail	*Caelorinchus innotabilis*	3 (25)	0.138±0.091	9.5±6.1
	Globosehead Whiptail	*Cetonurus globiceps*	1 (8.3)	0.017±0.017	1.9±1.9
	Little Whiptail	*Coelorinchus gormani*	2 (16.7)	1.379±0.940	48.6±32.9
	Serrulate Whiptail	*Coryphaenoides serrulatus*	3 (25)	0.242±0.172	35.6±25.7
	Toothed Whiptail	*Lepidorhynchus denticulatus*	3 (25)	1.492±1.265	81.3±68.3
	Whiptail	Macrouridae sp. 1	2 (16.7)	0.988±0.909	26.9±25.9
	Whiptail	Macrouridae sp. 2	2 (16.7)	0.017±0.013	2.3±1.8
	Whiptail	Macrouridae sp. 3	1 (8.3)	0.004±0.004	0.7±0.7
	Whiptail	Macrouridae sp. 4	1 (8.3)	0.004±0.004	0.1±0.1
	Blackspot Whiptail	*Ventrifossa nigromaculata*	1 (8.3)	0.017±0.017	0.2±0.2
Macruronidae	Blue Grenadier	*Macruronus novaezelandiae*	1 (8.3)	0.038±0.038	7.5±7.5
Melanonidae	Pelagic Cod	*Melanonus gracilis*	2 (16.7)	0.058±0.041	0.6±0.4
Monacanthidae	Leatherjacket	*Eubalichthys* sp. 1	4 (33.3)	0.196±0.093	44±19.2
Moridae	Slender Cod	*Halargyreus johnsonii*	2 (16.7)	0.025±0.018	6.7±5.0
	Ribaldo	*Mora moro*	2 (16.7)	0.029±0.020	26.3±23.9
	Deepsea Cod	*Moridae* sp.	1 (8.3)	0.004±0.004	0.1±0.1
	Grenadier Cod	*Tripterophycis gilchristi*	2 (16.7)	0.025±0.018	0.3±0.2
Myctophidae	Myctophid	Myctophidae sp. 1	2 (16.7)	0.067±0.049	0.1±0.1
	Myctophid	Myctophidae sp. 2	1 (8.3)	0.004±0.004	0.1±0.1
Neosebastidae	Gurnard Perch	*Neosebastes pandus*	1 (8.3)	0.004±0.004	1.0±1.0
	Ruddy Gurnard Perch	*Neosebastes scorpaenoides*	3 (25)	0.063±0.033	30.4±16.4
	Thetis Fish	*Neosebastes thetidis*	3 (25)	0.029±0.018	12.8±7.7
Ophidiidae	Pink Ling	*Genypterus blacodes*	1 (8.3)	0.004±0.004	8.3±8.3
Oplegnathidae	Knifejaw	*Oplegnathus woodwardi*	1 (8.3)	0.008±0.008	3.8±3.8
Oreosomatidae	Warty Oreo	*Allocyttus verrucosus*	3 (25)	0.308±0.204	106.8±80.2
	Spiky Oreo	*Neocyttus rhomboidalis*	1 (8.3)	0.008±0.008	3.5±3.5
Paraulopidae	Cucumber Fish	*Chlorophthalmus nigripinnis*	1 (8.3)	0.008±0.008	1.2±1.2
Pentacerotidae	Giant Boarfish	*Paristiopterus labiosus*	1 (8.3)	0.004±0.004	9.2±9.2
	Long Snouted Boarfish	*Pentaceropsis recurvirostris*	1 (8.3)	0.004±0.004	3.3±3.3
Platycephalidae	Toothy Flathead	*Neoplatycephalus aurimaculatus*	1 (8.3)	0.071±0.071	79.8±79.8
	Tiger Flathead	*Neoplatycephalus richardsoni*	3 (25)	0.054±0.030	17.4±10.4
	Sand Flathead	*Platycephalus bassensis*	3 (25)	0.113±0.091	82.1±59.6
Pristiophoridae	Southern Sawshark	*Pristiophorus nudipinnis*	1 (8.3)	0.004±0.004	6.4±6.4
Rajidae	Deepwater Skate	*Raja* sp. 3	3 (25)	0.013±0.007	0.8±0.5
Sebastidae	Ocean Perch	*Helicolenus percoides*	2 (16.7)	0.046±0.038	10.3±8.4
Somniosidae	Golden Dogfish	*Centroscymnus crepidater*	2 (16.7)	0.021±0.014	9.8±7.4
	Owstons Dogfish	*Centroscymnus owstoni*	1 (8.3)	0.004±0.004	13.4±13.4
Squalidae	Spikey Dogfish	*Squalus megalops*	4 (33.3)	0.029±0.014	24.1±12.1
Squatinidae	Angel Shark	*Sqatina australis*	1 (8.3)	0.033±0.033	72.1±72.1
Synaphobranchidae	Grey Cuthroat Eel	*Synaphobranchus affinis*	2 (16.7)	0.046±0.041	23.4±20.4
	Eel	*Synaphobranchus* sp. 1	1 (8.3)	0.017±0.017	3.4±3.4
	Eel	*Synaphobranchus* sp. 2	1 (8.3)	0.004±0.004	2.9±2.9
Trachichthyidae	Orange Roughy	*Hoplostethus atlanticus*	2 (16.7)	0.092±0.087	26.0±23.8
	Sandpaper Fish	*Paratrachichthys* sp.	1 (8.3)	0.004±0.004	0.7±0.7
Triakidae	Gummy Shark	*Mustelus antarcticus*	1 (8.3)	0.004±0.004	10.2±10.2
Triglidae	Minor Gurnard	*Lepidotrigla modesta*	5 (41.7)	0.700±0.424	121.3±76.8
	Butterfly Gurnard	*Lepidotrigla vanessa*	4 (33.3)	0.079±0.043	37.7±21.9
	Latchet	*Pterygotrigla polyommata*	6 (50)	0.621±0.250	235.0±99.3
Uranoscopidae	Speckled Stargazer	*Kathetostoma canaster*	5 (41.7)	0.046±0.020	127.3±50.2
	Deepwater Stargazer	*Kathetostoma nigrofasciatum*	1 (8.3)	0.004±0.004	0.8±0.8
Urolophidae	Banded Stingaree	*Urolophus cruciatus*	4 (33.3)	0.050±0.027	24.1±17.4
	Sparesly Spotted Stingaree	*Urolophus paucimaculatus*	3 (25)	0.071±0.038	39.9±22.3

The latchet *Pterygotrigla polyommata* was the most widespread fish encountered around the Bonney Canyon, and was collected at 50% (6/12) of the sites surveyed. Two other species (the minor gurnard *Lepidotrigla modesta* and the speckled stargazer *Kathetostoma canister*) were also broadly distributed and were found at 42% (5/12) of the trawl sites. Most species, by comparison, had restricted distributions and were found at less than 33% (4/12) of the survey sites. Indeed, almost half of all species collected (38/78) were only encountered at a single trawl site.

Few fish could be considered particularly abundant and only three species (the whiptails *Lepidorhynchus denticulatus*, *Coelorinchus gormani*, and Macrouridae sp. 1) were found at average densities of 1 or more per hectare (Table 1). The majority of fish (68% of species) had overall densities ranging from 0.01 to 1 per hectare, while the remaining 28% of species were present in densities of less than 0.01 per hectare. This later group of relatively rare species included the broadnose seven gill shark *Notorynchus cepedianus*, Owston's dogfish *Centroscymnus owstoni*, the southern sawshark *Pristiophorus nudipinnis*, Plunket's shark *Centroscymnus plunketi*, the giant boarfish *Paristiopterus labiosus*, and the deepwater stargazer *Kathetostoma nigrofasciatum*.

Not only was the latchet *Pterygotrigla polyommata* the most widely distributed species, this fish was also dominant in terms of biomass, and accounted for 11% of the total catch (mean biomass = 235.0 g/ha). A further five species individually contributed more than 5% to the overall standing-stock. These included, in order of descending biomass, the jackass morwong *Nemadactylus macropterus* (196.6 g/ha), the speckled stargazer *Kathetostoma canister* (127.3 g/ha), the minor gurnard *Lepidotrigla modesta* (121.3 g/ha), the redbait *Emmelichthys nitidus* (111.1 g/ha), and the warty oreo *Allocyttus verrucosus* (106.8 g/ha). All other species (92%) had mean biomasses ranging from 0.1 to 82.3 g/ha, and individually contributed less than 4% to the overall catch.

### Spatial patterns in richness and biomass

**Figure 6:**
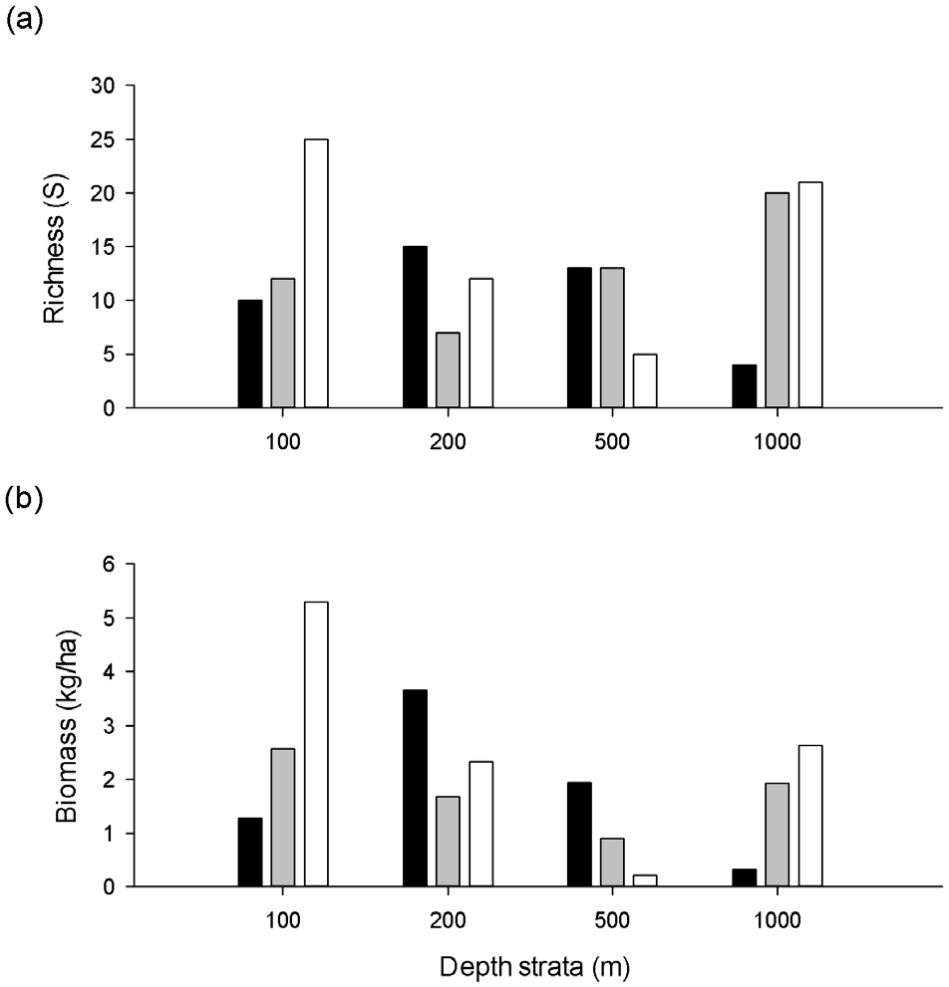
Bar graphs of demersal fish richness and biomass collected from trawl shots at 4 depth strata (100 m, 200 m, 500 m, 1000 m) around the head of Bonney Canyon. Locations relative to the main canyon axis are represented by different shades of fill (black = west, grey = centre, white = west).

Species richness (i.e. number of species per trawl) and biomass were highly correlated (Table 2) and the spatial patterns of richness and biomass were broadly similar (Figures 6a–b). The highest fish biomasses (3.7–5.3 kg/ha) were found on the shelf, and in particular at a depth of 100 m to the east, and 200 m to the west, of the central canyon axis. Moderately high measures of standing-stock (1.9–2.6 kg/ha) were also recorded from 1000 m depth on the central canyon axis and to the east of the canyon, but were generally low elsewhere on the slope. Similarly, species richness was highest on the shelf (25 species) at a depth of 100 m to the east of the central canyon axis. Relatively large numbers of fish species (20–21) were also collected from 1000 m depth on the central canyon axis and to the east of the canyon. ANOVA tests show that there are no significant differences in either fish biomass or richness on and either side of the central canyon axis and among the different depth strata (Table 3). It should, however, be noted that the statistical power associated with these tests was low (<0.1).

**Table 2:** Pearson correlation coefficients between species richness and biomass of demersal fish and adjacent environmental conditions at 12–15 depth stratified sampling sites around the Bonney Canyon.

	Depth	Temperature	Salinity	Oxygen	Chlorophyll	% Mud	Sediment Sorting	Sediment Carbon	Sediment Nitrogen	Richness
Depth	.	.	.	.	.	.	.	.	.	.
Temperature	−0.970**	.	.	.	.	.	.	.	.	.
Salinity	−0.685**	0.812**	.	.	.	.	.	.	.	.
Oxygen	−0.972**	0.976**	0.681**	.	.	.	.	.	.	.
Chlorophyll	−0.399	0.445	0.629	0.316	.	.	.	.	.	.
% Mud	0.934**	−0.968**	−0.772**	−0.971**	−0.376	.	.	.	.	.
Sediment Sorting	0.726**	−0.784**	−0.545*	−0.825**	−0.030	0.802**	.	.	.	.
Sediment Carbon	0.775**	−0.848**	−0.794**	−0.829**	−0.363	0.917**	0.772**	.	.	.
Sediment Nitrogen	0.830**	−0.868**	−0.793**	−0.835**	−0.304	0.899**	0.774**	0.940**	.	.
Richness	0.047	−0.027	0.104	−0.162	0.481	0.120	0.344	0.238	0.137	.
Biomass	−0.393	0.411	0.568	0.211	0.714**	−0.271	0.048	−0.216	−0.336	0.797**

Significant correlations after Bonferroni correction are denoted at the **1% level and *5% level.

**Table 3:** Results of two-way ANOVA tests for differences in richness and biomass of demersal fish taken in trawls shots at four strata (depth) on three transects located on and either side of the central canyon axis (region).

Dependent	Source	df	MS	F	*P*	Power
Richness	Region	1	0.042	0.001	0.982	0.050
	Depth	3	22.375	0.305	0.822	0.075
	Region* Depth	3	32.153	0.438	0.738	0.087
	Error	4	73.375			
Biomass	Region	1	0.503	0.154	0.715	0.061
	Depth	3	1.856	0.569	0.665	0.099
	Region * Depth	3	0.381	0.117	0.946	0.060
	Error	4	3.262			

Fish biomass increased significantly in relation to the chlorophyll concentration, but the closely allied measure of species richness was uncorrelated with this proxy for primary production (Table 2). All other environmental variables measured in this study (i.e. depth, temperature, salinity, oxygen concentration, % mud, sediment sorting, sediment carbon and sediment nitrogen) co-varied with one-another, but species richness or biomass were not correlated with these variables. In contrast to biomass and richness, community structure varied strongly with depth.

### Community structure

A PERMANOVA test was applied to evaluate the statistical significance of any canyon or depth-related differences in demersal fish community structure (Table 4). This test showed that fish assemblages did not vary significantly between the central canyon axis and the adjacent slope and shelf. However, faunal composition was found to vary significantly with depth. A *post hoc* pair-wise test (PERMANOVA, α = 0.05) confirmed that the demersal fish community structure differed significantly between all but the two shallowest depth strata (i.e. 100 m = 200 m≠500 m≠1000 m).

**Table 4:** Results of two-way PERMANOVA test for differences in demersal fish community structure between four strata (depth) on three transects located on and either side of the central canyon axis (region).

Source	df	MS	Pseudo-F	*P*	Permutations
Region	1	1603.8	0.645	0.722	3610
Depth	3	8792.4	3.537	0.002	9883
Region*Depth	3	1453.0	0.585	0.931	9873
Error	4	2485.5			

**Figure 7:**
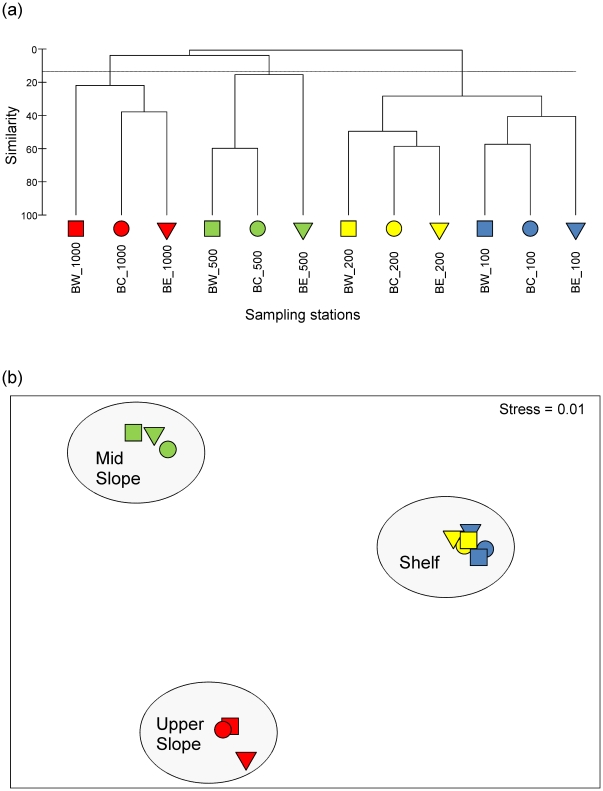
Dendrogram and non-metric MDS ordination of demersal fish community structure at 12 depth-stratified sampling stations located around the head of Bonney Canyon. Transects relative to the main canyon axis are represented by different symbols (squares = west, circles = centre, triangles = east), and depths by different shades of fill (white = 100 m, light grey = 200 m, dark grey = 500 m, black = 1000 m). Three station groupings are identified from the SIMPROF permutation test at the 5% significance level (dotted line): shelf, upper slope and mid slope.

Three discrete station groupings consistent with the PERMANOVA result were identified from MDS and cluster analyses using the SIMPROF permutation test at the 5% significance level (Figure 7). These included a “shelf” group comprising all 6 stations surveyed at 100 m and 200 m depth, an “upper slope” group containing all 3 trawl stations at 500 m depth, and a “mid slope” group consisting of all 3 stations surveyed at 1000 m depth.

SIMPER analysis was undertaken to determine which taxa contributed most to similarities within, and differences between, the three station groupings. Biomasses of the 14 species contributing ≥5% to within-group similarity or between-group dissimilarity for at least one of the three groupings are given in Table 5. Results from the SIMPER analysis indicate that all station groupings are characterised by small subsets of species.

**Table 5:** Mean biomass (g per ha ± s.e.) of fish species in three station groups identified from MDS classification.

Common Name	Scientific Name	Shelf (*n* = 6)	Upper Slope (*n* = 3)	Mid Slope (*n* = 3)
Toothed Whiptail	*Lepidorhynchus denticulatus*		300.7±264.1	24.7±24.7
Little Whiptail	*Coelorinchus gormani*		**194.3±97.9**	
Minor Gurnard	*Lepidotrigla modesta*	242.5±141.7		
Latchet	*Pterygotrigla polyommata*	**470.0±146.0**		
Warty Oreo	*Allocyttus verrucosus*			**427.3±270.0**
Jackass Morwong	*Nemadactylus macropterus*	**393.2±211.2**		
Serrulate Whiptail	*Coryphaenoides serrulatus*			**142.3±83.3**
Banded Bellowfish	*Centriscops humerosus*		46.0±23.1	
Leatherjacket	*Eubalichthys* sp. 1	88.0±29.1		
Notable Whiptail	*Caelorinchus innotabilis*			38.0±16.5
King Dory	*Cyttus traversi*		**166.0±79.7**	
Conger Eel	*Bassanago* sp. 1		88.0±65.1	30.0±15.5
Speckled Stargazer	*Kathetostoma canaster*	**254.7±67.8**		
Brier Shark	*Deania calcea*			284.0±284.0

Species listed were identified from SIMPER analyses as contributing ≥5% to the similarity within and dissimilarity between regional groupings. Those species indicative of each regional grouping (contributing ≥10% to the total similarity within a group) are highlighted in bold. Species are ranked in order of decreasing abundance across all station groupings.

The “shelf” group was the most diverse and consisted of 36 species, 33 (92%) of which were found only at stations located in depths of 200 m or less. Three species representing three families typified this group, and contributed more than 10% to the within-group similarity. These included the latchet *Pterygotrigla polyommata*, the jackass morwong *Nemadactylus macropterus*, and the speckled stargazer *Kathetostoma canister*.

The “upper slope” group was the least diverse and was composed of 19 species. Of these, 14 (74%) where unique to stations sampled at 500 m depth. This group was characterised by two species, notably the little whiptail *Coelorinchus gormani* and the king dory *Cyttus traversi*. These locally common species collectively accounted for 35% of the total group biomass, and 74% of the within-group similarity.

The “mid slope” group contained 29 species, 26 (90%) of which were found exclusively at stations located in depths of 1000 m. Two species, the warty oreo *Allocyttus verrucosus* and the serrulate whiptail *Coryphaenoides serrulatus*, dominated the catches from this depth and accordingly characterised this station grouping.

### Environmental linkages to community structure

The PRIMER routine BIOENV was used to assess the correspondence and significance of environmental data from the seafloor to the three station groupings identified from the community analyses. Three environmental variables (i.e. salinity, oxygen concentration and % mud) were excluded prior to conducting this analysis because they were highly correlated with depth (0.9>r<−0.9). Depth was correlated most closely with the community structure (*ρw* = 0.78). The other variables were individually much more weakly correlated to the community structure (sediment carbon *ρw* = 0.64, sediment nitrogen *ρw* = 0.62, salinity *ρw* = 0.60, sediment sorting *ρw* = 0.29, chlorophyll concentration *ρw* = 0.02). We conclude that variables other than depth failed to provide an improved explanation for the biological pattern.

## Discussion

**Figure 8:**
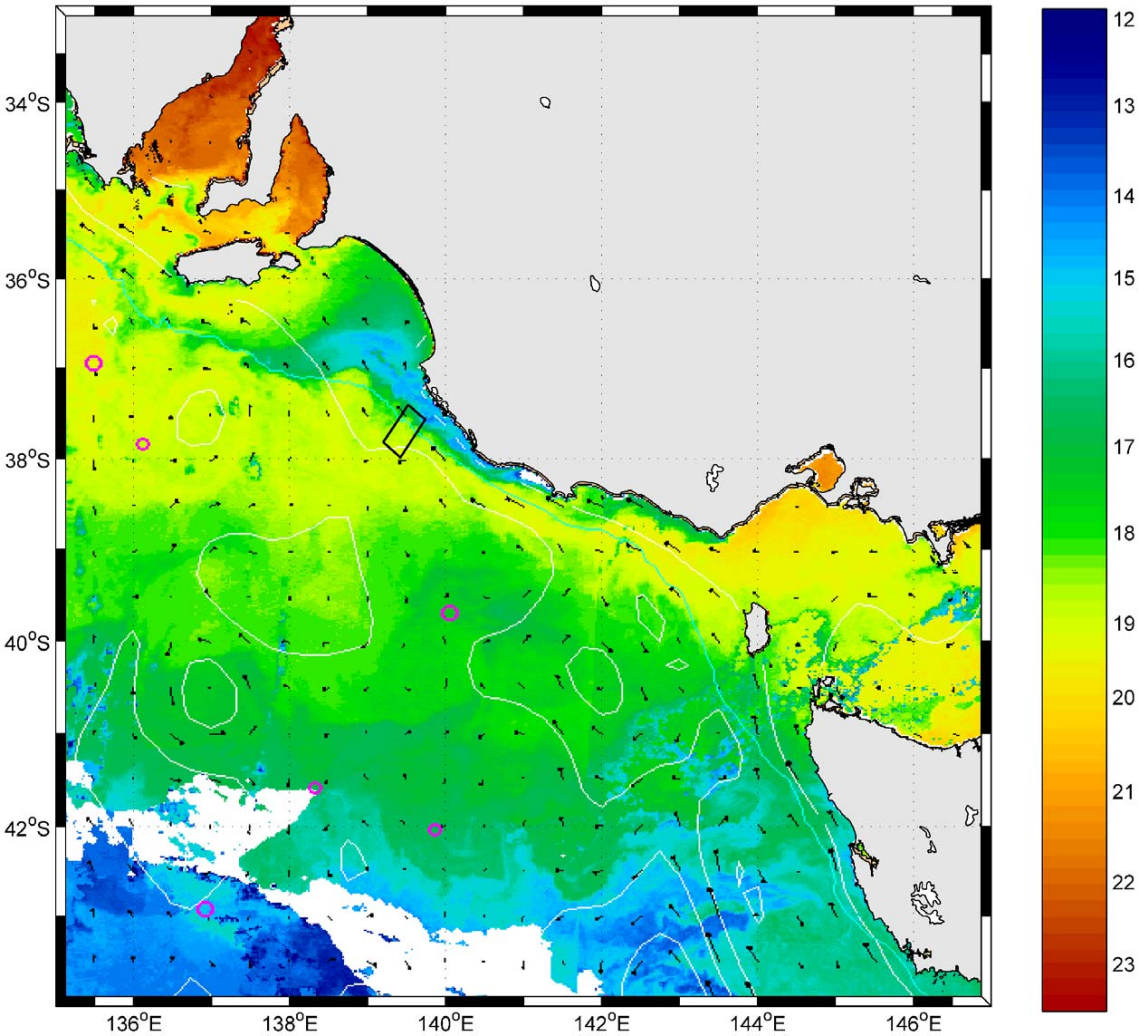
Sea surface temperature (SST °C) map recoded by NOAA satellite during voyage SS02/2008 showing an area of intense upwelling straddling the Bonney Canyon study area (unfilled rectangle). Image courtesy of CSIRO Marine and Atmospheric Research.

This voyage was scheduled to coincide with the most favourable period for upwelling (early February), and fortuitously our sampling coincided with one of the most significant upwelling events recorded off South Australia. Satellite SST imagery of this event confirmed that the upwelling extended along most of the Bonney Coast, and at least for 50 km either side of the Bonney Canyon (Figure 8). Indeed, the upwelling was the strongest on record, as evidenced by bottom temperatures on the 50 m isobath of three standard deviations below the long-term mean, indicating a one-in-one-hundred-year event.

Our conceptual model for upwelling and mixing for the region suggests that wind-forced upwelling in the near-shore zone is very strong, and probably active over the shelf (100–200 m). It also indicates that canyon upwelling can occur, but this appears to have been arrested through thermal wind shear during our study. Furthermore, the upwelled isopycnals at depths of 600–900 m are probably a reflection of the bottom boundary layer of the Flinders Current. Salt fingering appears to be a likely explanation for the strong degree of water mixing observed below the surface mixed layer (50–75 m) to depths of 450 m.

We hypothesise that there exists some form of instability that is able to mix water between the surface mixed layer and depths of 600–900 m. The buoyancy frequency plot of N^2^ (not shown) indicates that there are large regions where the water column is statically unstable (N^2^<0). The plot of density versus depth (Figure 3) also indicates regions where the vertical density gradient is zero or negative. While internal waves might be responsible, it is also possible that the instabilities and mixing result from double diffusive salt fingers. Such fingers can result where hot, salty water lies over cool, fresh water. This is because heat diffuses faster than salt, and a descending hot salty finger can become cooler leading to it being denser than its surroundings.

Although there is compelling oceanographic evidence for canyon-related upwelling around Bonney Canyon, our biological data showed no clear trends in relation to the canyon orientation or topography. For example, neither biomass nor species richness of demersal fishes was significantly different between the central canyon axis and the adjacent shelf and slope. In addition, we found no evidence for any change in the composition of species represented on and either side of the central canyon axis. Unfortunately there is little empirical evidence available to assess whether these patterns in demersal fish at Bonney Canyon are typical of submarine canyons more generally. Many research studies highlight the importance of canyons in focusing primary productivity (e.g. [4], [37], [38], [39], [40], [41]), however flux to the benthos and trophic linkages with demersal fauna remain poorly understood [42], [43], [44]. To date, only a small number of studies have directly compared the biomass and composition of demersal fish assemblages between the central axis of a submarine canyon and the adjacent slope [2], [45], [46]. Stefanescu *et al.* [46] working on the continental slope of the Mediterranean Sea, observed that demersal fish abundances and biomasses were much higher inside the Rec del Besos Canyon than on other parts of the adjacent slope. These researchers attributed the differences in standing-stock to elevated trophic resources inside the canyon. They also reported that the canyon supported smaller-sized individuals of several common species, and suggested that the canyon was a recruiting ground for many of these. However, as in our study, they could not detect any canyon-related differences in species composition.

Most benthic communities at depths below the photic zone are dependent on sinking water column production as a major source of food, hence the quality and quantity of organic matter reaching the seafloor is an important influence on benthic community structure and biomass [47], [48], [49]. While hotspots of demersal fish diversity may be highly correlated with regions of enhanced surface phytoplankton concentrations (e.g. along the margins of the Chatham Rise, east of New Zealand [50]), standing-stock and surface production data are not always concurrent [51]. Horizontal advection can complicate this linkage through the transport of sinking phytoplankton to a bottom area that is distant from the surface waters where they were abundant [52]. Decoupling between herbivory and primary production can further modify the export of pelagic production to the benthos as a result of changes in zooplankton grazing rates [53]. The extent to which such factors influence demersal fish distributions around Bonney Canyon are uncertain. However it is notable that the fish biomasses were generally higher on the shallow shelf waters where benthic chlorophyll levels were elevated.

Whilst no clear canyon-related differences in demersal fish community structure were detected around Bonney Canyon, marked differences in community structure were observed in relation to depth. Such bathymetric changes in community structure are widely reported on shelf and slope habitats [54], [55], [56], [57], [58], but geographical differences between studies, as well as variations in the range of depths considered or the classification methods employed, mean that patterns are often contradictory. In our study spanning the shelf and slope (100–1000 m) off southern Australia, three distinct communities were identified. These results are broadly consistent with Koslow *et al.* [59], who examined trawl data from a narrower and deeper range off southeastern Australia (500–1200 m), and identified distinct assemblages in the upper (500 m) and mid slope (800–1200 m).

Koslow *et al.* [59], in recognising affinities between the southeast Australian mid slope fish communities and those at similar depths in the North Atlantic, suggested that biogeographic patterns were consistent with ocean circulation at intermediate depths. Notably, they observed that their mid slope community resided within the core depth range (800–1200 m) of the Antarctic Intermediate Water mass, which extends around the northern rim of the Southern Ocean [60]. Like Koslow e*t al.* [59], our mid slope community also corresponds with Antarctic Intermediate Water, but faunal discontinuities at the upper slope and shelf also coincide with our estimates of the upper and lower vertical boundaries (450–900 m) of the westward moving Flinders Current. Similar zonational patterns in demersal fish are also reported on the West Australian continental slope, where community breaks at 300 m and 700 m depth coincide with lower boundary limits of the near-surface Leeuwin Current and upper extent of the Antarctic Intermediate Water, respectively. While such correlations are not necessarily causative, they are intriguing, and suggest that ocean circulation patterns play an important role in structuring fish communities at regional scales (10–1000 km). More intensive depth-stratified sampling across the shelf and slope will undoubtedly assist us in identifying potential zones of transition in community structure, and their prospective drivers.

In our community analyses, depth was identified as the most import factor structuring fish assemblages around Bonney Canyon. However, depth is unlikely be the primary causal factor determining faunal composition. This is because many other physical/chemical variables co-vary with depth (e.g. temperature, salinity, oxygen) and may also influence the distribution of benthic species. Water circulation patterns for example, which may also vary with depth, can influence benthic communities in several ways. In particular, water circulation can modify other water column processes, such as near-bottom flow, that bring food and new recruits to the community [61]. They can also influence the physical heterogeneity of the seafloor through processes of erosion and deposition, and thereby directly influence the distribution of habitats and associated species [62], [63]. A range of other biological processes (e.g. predation, competition) are also likely to have important influences on the distribution and abundance of demersal fish, particularly at local scales (1–1000 m), but remain unmeasured.

Although the 78 fish species collected around Bonney Canyon represent only a small component of the total Australian fauna (>4500 species [64]), the species richness is relatively high when compared with other shelf and slope environments. For example, a total of 39 species of demersal fish were collected from more than twice as many trawls (32) off Newfoundland in the North Atlantic (204–2325 m) [54]. Relatively few species (44) were also collected from a similar number of trawls (35) in the western Mediterranean (350–1300 m) [46]. While in a survey off Prince Edward Archipelago in the Southern Ocean, 54 putative species were collected from 52 trawls (200–1500 m) [65]. On the basis of these preliminary comparisons it is tempting to suggest that demersal fish diversity around Bonney Canyon is globally high. However, such comparisons are invariably confounded by several factors including differences between studies in the range and area of habitats surveyed, and the types of trawl gear employed.

Comparisons of fish diversity between studies are further complicated by the history and intensity of human impacts, such as demersal fishing. A number of reviews highlight the fact that demersal fishing gears such as beam-trawls, otter trawls and dredges modify benthic habitats and fauna [66], [67], [68]. While fishing directly affects the structure of fish communities by reducing the abundance of the target species, these reductions can also have important indirect effects on the community structure mediated by modified competitive interactions and predator-prey relationships [67]. The time scales over which fishery induced community changes develop are also likely to vary with depth, as many deepwater species have life-history characteristics that differ from shelf species (i.e. increased longevity, slower growth rates and late maturation [69]). Demersal trawling has been ongoing in the offshore waters of southeastern Australia for almost 100 years, and it is estimated that at least 65% of the upper slope and a large portion of the mid slope have been trawled in recent years [70]. While the cumulative effects of historical trawling impacts on the demersal fish assemblages around Bonney Canyon are unknown, it appears that trawling is becoming increasingly concentrated in Australia's southern canyons, due in part to improvements in navigational technologies [70], [71]. We therefore cannot discount the possibility that patterns in fish distribution observed in this study reflect to some degree the persistent effects of historical fishing.

## Supporting Information

Table S1 **List of sampling operations undertaken around Bonney Canyon during Southern Surveyor voyage SS02/2008.** Method codes denote: [1] trawl shots excluded due to hook-up or over-spreading, [2] grab misfire, [3] repeat CTD cast for productivity measurement.
(DOC)

Table S2 **Summary of seabed properties at 15 depth-stratified survey sites located around the head of Bonney Canyon.** Station labels denote location relative to the central canyon axis (BW = Bonney West, BC = Bonney Centre, BE = Bonney East) and sampling depth strata in metres (100, 200, 500, 1000, 1500).
(DOC)
