# Supplementary material for: Biophysical Factors Affecting the Distribution of Demersal Fish around the Head of a Submarine Canyon Off the Bonney Coast, South Australia
Source: PLoS One. 2012 Jan 11;7(1):e30138. doi: 10.1371/journal.pone.0030138 (PMC3256224; doi:10.1371/journal.pone.0030138)
Supplement: Table S1 — List of sampling operations undertaken around Bonney Canyon during Southern Surveyor voyage SS02/2008. Method codes denote: [1] trawl shots excluded due to hook-up or over-spreading, [2] grab misfire, [3] repeat CTD cast for productivity measurement. (DOC) [file pone.0030138.s001.doc]

**Table S1**. List of sampling operations undertaken around Bonney Canyon during Southern Surveyor voyage SS02/2008. Method codes denote: [1] trawl shots excluded due to hook-up or over-spreading, [2] grab misfire, [3] repeat CTD cast for productivity measurement.

| **#** | **Site** | **Date** | **Method** | **Start**  **Time** | **Finish**  **Time** | **Start**  **Longitude** | **Start**  **Latitude** | **Finish Longitude** | **Finish**  **Latitude** | **Depth**  **(m)** |
| --- | --- | --- | --- | --- | --- | --- | --- | --- | --- | --- |
| 1 | BC_100 | 7/02/2008 | Trawl ^[1]^ | 07.34 | 07.37 | 139 34.924 | -37 28.651 | 139 36.737 | -37 30.132 | 92 |
| 2 | BC_100 | 7/02/2008 | CTD | 08.40 | 08.55 | 139 36.900 | -37 30.156 | - | - | 102 |
| 3 | BC_100 | 7/02/2008 | Grab | 09.06 | 09.12 | 139 36.944 | -37 30.151 | - | - | 103 |
| 4 | BW_100 | 7/02/2008 | CTD | 14.57 | 15.10 | 139 30.927 | -37 28.009 | - | - | 102 |
| 5 | BW_100 | 7/02/2008 | Grab | 15.20 | 15.25 | 139 31.291 | -37 28.026 | - | - | 104 |
| 6 | BE_100 | 7/02/2008 | CTD | 16.56 | 17.11 | 139 38.916 | -37.35.313 | - | - | 99 |
| 7 | BE_100 | 7/02/2008 | Grab | 17.20 | 17.24 | 139 39.438 | -37 35.546 | - | - | 100 |
| 8 | BE_200 | 8/02/2008 | CTD | 02.50 | 03.02 | 139 37.286 | -37 38.635 | - | - | 182 |
| 9 | BE_200 | 8/02/2008 | Grab | 03.09 | 03.15 | 139 37.156 | -37 38.630 | - | - | 192 |
| 10 | BC_200 | 8/02/2008 | CTD | 04.05 | 04.23 | 139 34.672 | -37 36.054 | - | - | 196 |
| 11 | BC_200 | 8/02/2008 | Grab | 04.30 | 04.37 | 139 34.410 | -37 36.026 | - | - | 200 |
| 12 | BW_200 | 8/02/2008 | CTD | 05.24 | 05.38 | 139 27.605 | -37 32.650 | - | - | 160 |
| 13 | BW_200 | 8/02/2008 | Grab | 05.44 | 05.54 | 139 27.419 | -37 32.665 | - | - | 160 |
| 14 | BW_200 | 8/02/2008 | Trawl | 07.21 | 07.51 | 139 27.279 | -37 31.373 | 139 27.133 | -37 32.760 | 155 |
| 15 | BC_200 | 8/02/2008 | Trawl | 09.28 | 09.58 | 139 33.559 | -37 33.685 | 139 34.144 | -37 34.825 | 147 |
| 16 | BE_200 | 8/02/2008 | Trawl | 11.15 | 11.45 | 139 36.719 | -37 37.114 | 139 37.485 | -37 38.414 | 150 |
| 17 | BE_500 | 8/02/2008 | CTD | 23.01 | 23.42 | 139 34.458 | -37 42.432 | - | - | 456 |
| 18 | BE_500 | 8/02/2008 | Grab | 23.54 | 00.07 | 139 34.486 | -37 42.438 | - | - | 456 |
| 19 | BC_500 | 9/02/2008 | CTD | 00.52 | 01.17 | 139 32.309 | -37 38.519 | - | - | 454 |
| 20 | BC_500 | 9/02/2008 | Grab | 01.25 | 01.38 | 139 32.311 | -37 38.523 | - | - | 450 |
| 21 | BW_500 | 9/02/2008 | CTD | 02.29 | 02.59 | 139 26.384 | -37 34.553 | - | - | 516 |
| 22 | BW_500 | 9/02/2008 | Grab | 03.04 | 03.18 | 139 26.365 | -37 34.570 | - | - | 520 |
| 23 | BW_500 | 9/02/2008 | Trawl ^[1]^ | 06.03 | 06.25 | 139 26.227 | -37 35.023 | 139 26.280 | -37 36.030 | 518 |
| 24 | BW_500 | 9/02/2008 | Trawl | 08.13 | 08.53 | 139 25.799 | -37 34.587 | 139 26.722 | -37 36.010 | 540 |
| 25 | BC_500 | 9/02/2008 | Trawl ^[1]^ | 11.20 | 11.35 | 139 31.150 | -37 37.822 | 139 31.752 | -37 38.248 | 520 |
| 26 | BE_1000 | 9/02/2008 | CTD | 21.53 | 22.23 | 139 30.779 | -37 47.700 | - | - | 992 |
| 27 | BE_1000 | 9/02/2008 | Grab | 22.41 | 23.09 | 139 30.771 | -37 47.698 | - | - | 992 |
| 28 | BC_1000 | 10/02/2008 | CTD | 00.01 | 00.45 | 139 28.994 | -37 41.589 | - | - | 951 |
| 29 | BC_1000 | 10/02/2008 | Grab | 00.54 | 01.19 | 139 28.983 | -37 41.615 | - | - | 952 |
| 30 | BE_500 | 10/02/2008 | Trawl ^[1]^ | 06.13 | 06.47 | 139 35.248 | -37 42.336 | 139 33.120 | -37 41.315 | 419 |
| 31 | BE_500 | 10/02/2008 | Trawl | 08.47 | 09.17 | 139 35.244 | -37 42.347 | 139 33.430 | -37 41.615 | 412 |
| 32 | BC_500 | 10/02/2008 | Trawl | 10.59 | 11.29 | 139 31.047 | -37 37.513 | 139 32.450 | -37 38.386 | 521 |
| 33 | BW_1000 | 10/02/2008 | CTD | 14.27 | 15.10 | 139 20.773 | -37 41.972 | - | - | 1001 |
| 34 | BW_1000 | 10/02/2008 | Grab | 15.20 | 15.51 | 139 20.853 | -37 41.991 | - | - | 1003 |
| 35 | BW_1500 | 10/02/2008 | CTD | 21.07 | 22.00 | 139 15.641 | -37 48.990 | - | - | 1503 |
| 36 | BW_1500 | 10/02/2008 | Grab | 22.13 | 23.00 | 139 15.643 | -37 49.021 | - | - | 1508 |
| 37 | BC_1500 | 11/02/2008 | CTD | 00.07 | 01.16 | 139 26.253 | -37 43.956 | - | - | 1597 |
| 38 | BC_1500 | 11/02/2008 | Grab | 01.29 | 01.58 | 139 26.209 | -37 43.957 | - | - | 1593 |
| 39 | BE_1500 | 11/02/2008 | CTD | 03.29 | 04.32 | 139 26.572 | -37 53.770 | - | - | 1504 |
| 40 | BE_1500 | 11/02/2008 | Grab ^[2]^ | 04.38 | 05.21 | 139 26.574 | -37 53.723 | - | - | 1501 |
| 41 | BE_1500 | 11/02/2008 | Grab | 05.27 | 06.00 | 139 26.544 | -37 53.818 | - | - | 1505 |
| 42 | BE_1000 | 11/02/2008 | Trawl | 08.01 | 08.53 | 139 30.077 | -37 46.640 | 139 31.080 | -37 48.368 | 1004 |
| 43 | BE_200 | 11/02/2008 | CTD ^[3]^ | 22.19 | 22.37 | 139 37.225 | -37 38.656 | - | - | 185 |
| 44 | BC_200 | 11/02/2008 | CTD ^[3]^ | 23.11 | 23.28 | 139 34.681 | -37 36.034 | - | - | 190 |
| 45 | BW_200 | 12/02/2008 | CTD ^[3]^ | 00.11 | 00.30 | 139 27.860 | -37 32.705 | - | - | 158 |
| 46 | BW_100 | 12/02/2008 | Trawl | 04.31 | 05.05 | 139 32.099 | -37 25.782 | 139 32.555 | -37 27.546 | 92 |
| 47 | BC_100 | 12/02/2008 | Trawl | 07.03 | 07.37 | 139 35.088 | -37 28.590 | 139 36.301 | -37 30.195 | 96 |
| 48 | BE_100 | 12/02/2008 | Trawl | 09.38 | 10.08 | 139 42.113 | -37 33.967 | 139 42.933 | -37 35.608 | 78 |
| 49 | BW_1000 | 13/02/2008 | Trawl | 03.02 | 03.29 | 139 18.670 | -37 40.541 | 139 20.106 | -37 41.969 | 1035 |
| 50 | BC_1000 | 13/02/2008 | Trawl | 06.35 | 07.20 | 139 26.291 | -37 38.820 | 139 27.874 | -37 40.246 | 949 |
| 51 | BC_100 | 14/02/2008 | Sled | 00.19 | 00.34 | 139 35.220 | -37 28.991 | 139 35.490 | -37 29.190 | 87 |
| 52 | BC_200 | 14/02/2008 | Sled | 01.30 | 01.45 | 139 33.965 | -37 34.211 | 139 34.081 | -37 34.491 | 152 |
| 53 | BC_500 | 14/02/2008 | Sled | 02.44 | 02.59 | 139 32.116 | -37 38.144 | 139 32.442 | -37 38.356 | 438 |
| 54 | BC_1000 | 14/02/2008 | Sled | 04.30 | 05.00 | 139 27.706 | -37 40.562 | 139 28.023 | -37 40.863 | 969 |
| 55 | BC_1500 | 14/02/2008 | Sled | 07.10 | 07.40 | 139 25.938 | -37 43.279 | 139 26.463 | -37 43.764 | 1523 |
| 56 | BC_2000 | 14/02/2008 | Sled | 09.42 | 10.35 | 139 23.530 | -37 48.220 | 139 23.945 | -37 49.440 | 1917 |
| 57 | PD10 | 15/02/2008 | Grab | 13.11 | 14.35 | 139 17.186 | -38 29.513 | - | - | 4125 |
| 58 | PD11 | 15/02/2008 | Grab | 15.09 | 16.39 | 139 18.884 | -38 28.476 | - | - | 4079 |
| 59 | PD13 | 15/02/2008 | Rock Dredge | 21.07 | 22.50 | 139 21.814 | -38 23.783 | 139 18.064 | -38 22.449 | 3392 |
| 60 | PC3 | 16/02/2008 | Beam Trawl | 03.40 | 04.15 | 139 19.750 | -37 52.480 | 139 20.600 | -37 53.390 | 2010 |
| 61 | BC_1000 | 16/02/2008 | Beam Trawl | 07.10 | 07.40 | 139 26.920 | -37 40.090 | 139 27.788 | -37 40.628 | 957 |
