# Supplementary material for: Biophysical Factors Affecting the Distribution of Demersal Fish around the Head of a Submarine Canyon Off the Bonney Coast, South Australia
Source: PLoS One. 2012 Jan 11;7(1):e30138. doi: 10.1371/journal.pone.0030138 (PMC3256224; doi:10.1371/journal.pone.0030138)
Supplement: Table S2 — Summary of seabed properties at 15 depth-stratified survey sites located around the head of Bonney Canyon. Station labels denote location relative to the central canyon axis (BW = Bonney West, BC = Bonney Centre, BE = Bonney East) and sampling depth strata in metres (100, 200, 500, 1000, 1500). (DOC) [file pone.0030138.s002.doc]

**Table S2**. Summary of seabed properties at 15 depth-stratified survey sites located around the head of Bonney Canyon. Station labels denote location relative to the central canyon axis (BW = Bonney West, BC = Bonney Centre, BE = Bonney East) and sampling depth strata in metres (100, 200, 500, 1000, 1500).

| Variable | BW_100 | BC_100 | BE_100 |  | BW_200 | BC_200 | BE_200 |  | BW_500 | BC_500 | BE_500 |  | BW_1000 | BC_1000 | BE_1000 |  | BW_1500 | BC_1500 | BE_1500 |
| --- | --- | --- | --- | --- | --- | --- | --- | --- | --- | --- | --- | --- | --- | --- | --- | --- | --- | --- | --- |
| Sediment size (%<63μ) | 1.23 | 0.97 | 2.34 |  | 1.90 | 2.04 | 1.82 |  | 13.52 | 5.93 | 12.46 |  | 56.07 | 54.43 | 52.57 |  | 56.34 | 56.61 | 48.45 |
| Sediment sorting (Phi) | 1.43 | 1.33 | 1.32 |  | 0.87 | 0.96 | 1.07 |  | 1.30 | 0.83 | 0.92 |  | 1.57 | 1.81 | 1.57 |  | 1.58 | 1.68 | 1.82 |
| Sediment carbon (% Organic) | 0.24 | 0.34 | 0.54 |  | 0.19 | 0.09 | 0.23 |  | 1.23 | 0.66 | 1.19 |  | 3.16 | 4.73 | 3.39 |  | 1.92 | 3.36 | 2.33 |
| Sediment nitrogen (%) | 0.04 | 0.06 | 0.04 |  | 0.01 | 0.01 | 0.01 |  | 0.09 | 0.06 | 0.09 |  | 0.13 | 0.16 | 0.13 |  | 0.11 | 0.16 | 0.12 |
| Temperature (°C) | 10.35 | 10.69 | 11.09 |  | 12.06 | 9.65 | 9.52 |  | 8.29 | 8.92 | 9.02 |  | 4.06 | 4.37 | 4.17 |  | 2.75 | 2.66 | 2.72 |
| Salinity | 34.86 | 34.91 | 34.98 |  | 35.14 | 34.74 | 34.73 |  | 34.55 | 34.63 | 34.64 |  | 34.40 | 34.39 | 34.39 |  | 34.57 | 34.60 | 34.57 |
| Oxygen (μM/l) | 253.88 | 252.69 | 251.53 |  | 254.73 | 249.91 | 253.59 |  | 241.04 | 249.67 | 251.98 |  | 190.45 | 194.15 | 192.73 |  | 171.07 | 170.68 | 171.04 |
| Chlorophyll (μg/l) | 11.22 | 13.87 | 14.28 |  | 11.13 | 10.33 | 9.97 |  | 9.92 | 10.34 | 9.93 |  | 9.72 | 10.05 | 10.70 |  | 10.95 | 10.73 | 9.97 |
